# Author Correction: Therapeutic manipulation of *IKBKAP* mis-splicing with a small molecule to cure familial dysautonomia

**DOI:** 10.1038/s41467-021-26287-8

**Published:** 2021-10-12

**Authors:** Masahiko Ajiro, Tomonari Awaya, Young Jin Kim, Kei Iida, Masatsugu Denawa, Nobuo Tanaka, Ryo Kurosawa, Shingo Matsushima, Saiko Shibata, Tetsunori Sakamoto, Lorenz Studer, Adrian R. Krainer, Masatoshi Hagiwara

**Affiliations:** 1 Department of Drug Discovery Medicine, Kyoto University Graduate School of Medicine, Kyoto, Japan; 2 Department of Anatomy and Developmental Biology, Kyoto University Graduate School of Medicine, Kyoto, Japan; 3 Cold Spring Harbor Laboratory, Cold Spring Harbor, NY USA; 4 Medical Research Support Center, Kyoto University Graduate School of Medicine, Kyoto, Japan; 5 Center for Stem Cell Biology, Sloan Kettering Institute, New York, NY USA

**Keywords:** Chemical biology, RNA splicing, Peripheral neuropathies

Correction to: *Nature Communications* 10.1038/s41467-021-24705-5, published online 23 July 2021.

In this article the author name Lorenz Studer was incorrectly written as Rolenz Studer. The original article has been corrected.

