# Supplementary material for: Therapeutic manipulation of IKBKAP mis-splicing with a small molecule to cure familial dysautonomia
Source: Nat Commun. 2021 Jul 23;12:4507. doi: 10.1038/s41467-021-24705-5 (PMC8302731; doi:10.1038/s41467-021-24705-5)
Supplement: Supplementary file 1 — supplementary information [file 41467_2021_24705_MOESM1_ESM.pdf]

Supplementary Information

Therapeutic manipulation of *IKBKAP* mis-splicing with a small molecule to cure familial dysautonomia

Supplementary Fig. 1. Treatment with RECTAS, CLK inhibitors, and SRPK inhibitors in primary fibroblasts from FD patient and healthy donor. (page 2)

Supplementary Fig. 2. Knockdown effect of SRSF6 and CLK isoforms on *IKBKAP* exon 20 splicing in primary fibroblasts from FD patient and healthy donor. (page 3)

Supplementary Fig. 3. Time-course experiment for exon 20 inclusion promoting activity by RECTAS. (page 4)

Supplementary Fig. 4. Diagram for human *IKBKAP* and mouse *Ikbkap*. (page 5)

Supplementary Fig. 5. RNA-Seq analysis for DRG from *IKBKAP*-FD transgenic mice treated with MOCK or RECTAS (300 mg/kg BW). (page 6)

Supplementary Fig. 6. Western blot analysis for knockdown efficiency. (page 7)

Supplementary Table 1. Summary of pre-clinical studies of RECTAS. (page 8)

Supplementary Table 2. List of primers used in this study. (page 9)

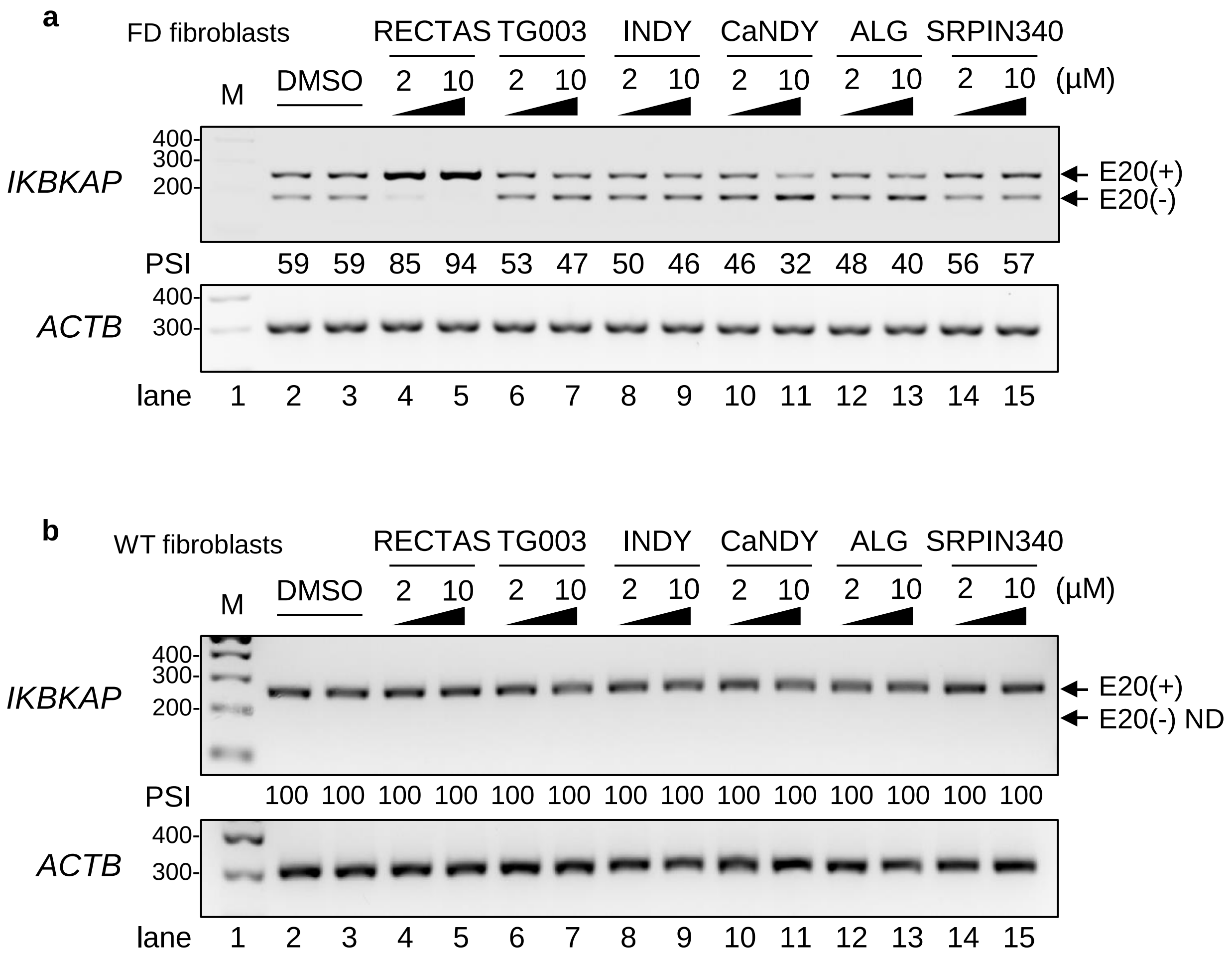

Supplementary Fig. 1. Treatment with RECTAS, CLK inhibitors, and SRPK inhibitors in primary fibroblasts from FD patient and healthy donor.

Compound response (24 h treatment at 2 or 10 µM) of endogenous *IKBKAP*-FD exon 20 was shown for primary fibroblasts from FD patient (P1) (a) and healthy donor (C2) (b). Solvent only (0.1% DMSO) served as a negative control, and *ACTB* served as a loading control. Representative data from two replicates are shown in (a) and (b). PSI, percent spliced-in; E20 (+) and E20 (-), exon 20 inclusion and skipping products, respectively. *IKBKAP* and *ACTB* were detected with primers oAM138+oAM139 and oAM13+oAM14, respectively.

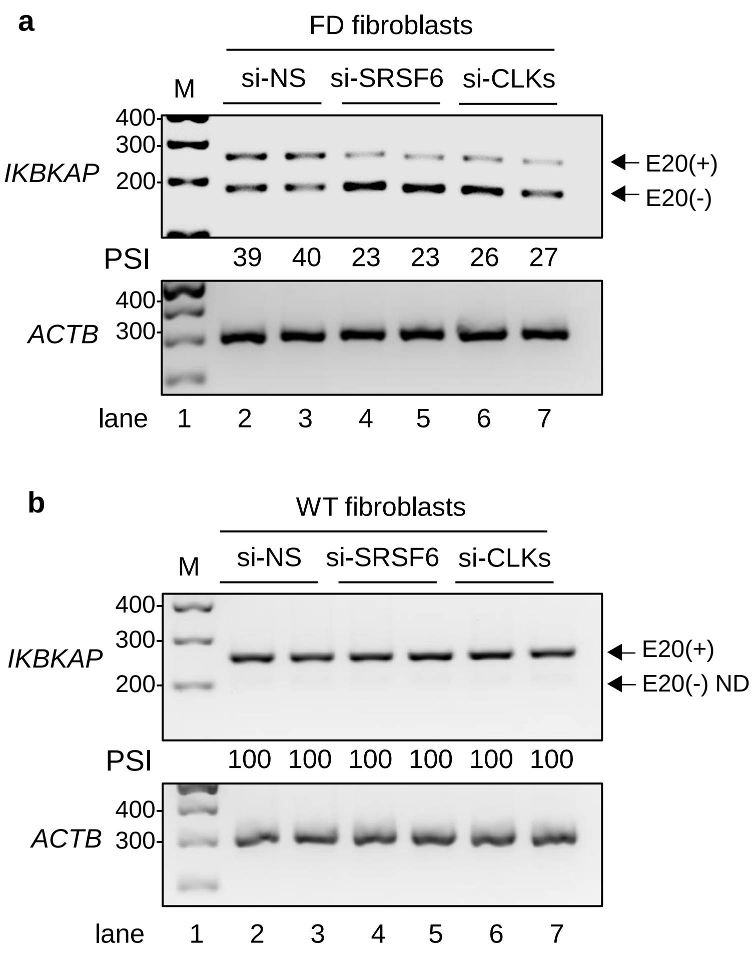

Supplementary Fig. 2. Knockdown effect of SRSF6 and CLK isoforms on *IKBKAP* exon 20 splicing in primary fibroblasts from FD patient and healthy donor.

Knockdown effect of SRSF6 (si-SRSF6) and CLK isoforms (si-CLKs) for endogenous *IKBKAP*-FD exon 20 was shown for primary fibroblasts from FD patient (P1) (a) and healthy donor (C2) (b). Non-targeting siRNA (si-NS) served as a negative control, and *ACTB* served as a loading control. Representative data from two replicates are shown in (a) and (b). PSI, percent spliced-in; E20 (+) and E20 (-), exon 20 inclusion and skipping products, respectively. *IKBKAP* and *ACTB* were detected with primers oAM138+oAM139 and oAM13+oAM14, respectively.

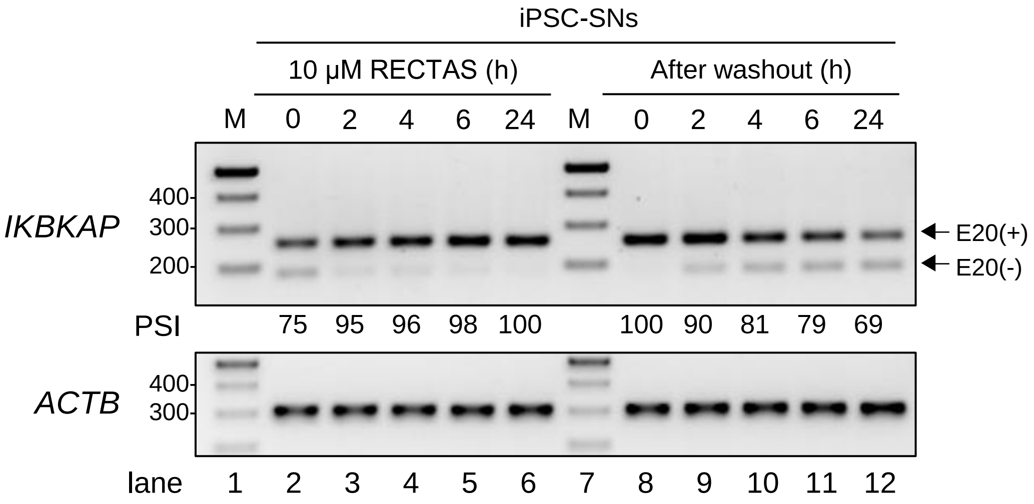

Supplementary Fig. 3. Time-course experiment for exon 20 inclusion promoting activity by RECTAS.

FD patient cell-derived iPSC-SNs at 12 days of induced differentiation were analyzed for *IKBKAP* exon 20 splicing at 0, 2, 4, 6, and 24 h after the RECTAS treatment (10 μM). 24 h-RECTAS treated iPSC-SNs were also subjected to washout by PBS (-) and kept in culture media without RECTAS, and analyzed at 0, 2, 4, 6, and 24 h after the washout. *ACTB* served as a loading control. Representative data from three replicates are shown. PSI, percent spliced-in; E20 (+) and E20 (-), exon 20 inclusion and skipping products, respectively. *IKBKAP* and *ACTB* were detected with primers oAM138+oAM139 and oAM13+oAM14, respectively.

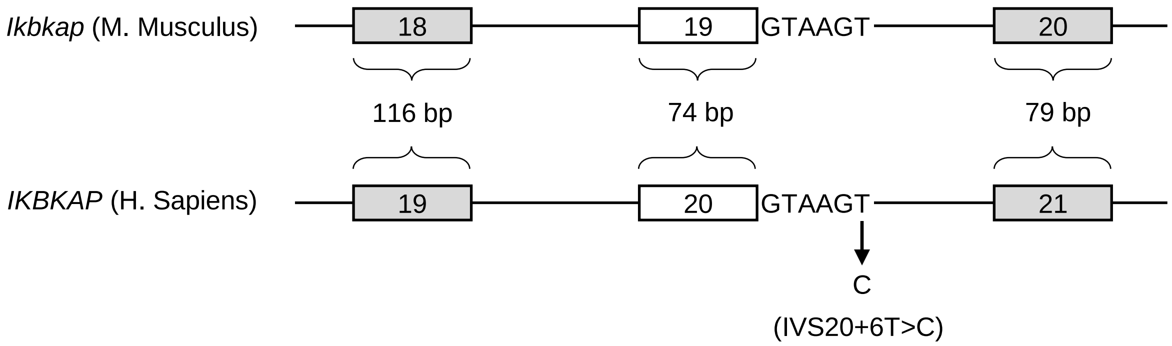

Supplementary Fig. 4. Diagram for human *IKBKAP* and mouse *Ikbkap*.

Exon 18, 19, and 20 of mouse *Ikbkap* is homologous to exon 19, 20, and 21 of human *IKBKAP* with matched exon length and surrounding intronic sequences.

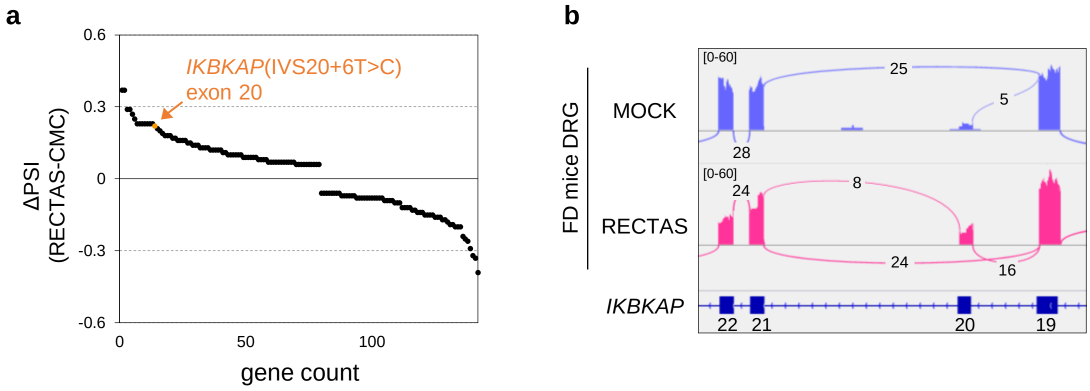

Supplementary Fig. 5. RNA-Seq analysis for DRG from *IKBKAP*-FD transgenic mice treated with MOCK or RECTAS (300 mg/kg BW).

(a) Differential splice events with >0.05 of |ΔPSI (PSI_{RECTAS}-PSI_{CMC})| in RECTAS (300 mg/kg BW)-administered mice over CMC-administered mice were plotted. Vertical, ΔPSI; Horizontal, gene count. Arrow (orange) indicates *IKBKAP*-FD exon 20 inclusion. (b) Representative sashimi plot with junction read number was shown for MOCK and RECTAS groups. *IKBKAP* gene structure is shown on the bottom. Displayed read-count range was set to 0-60.

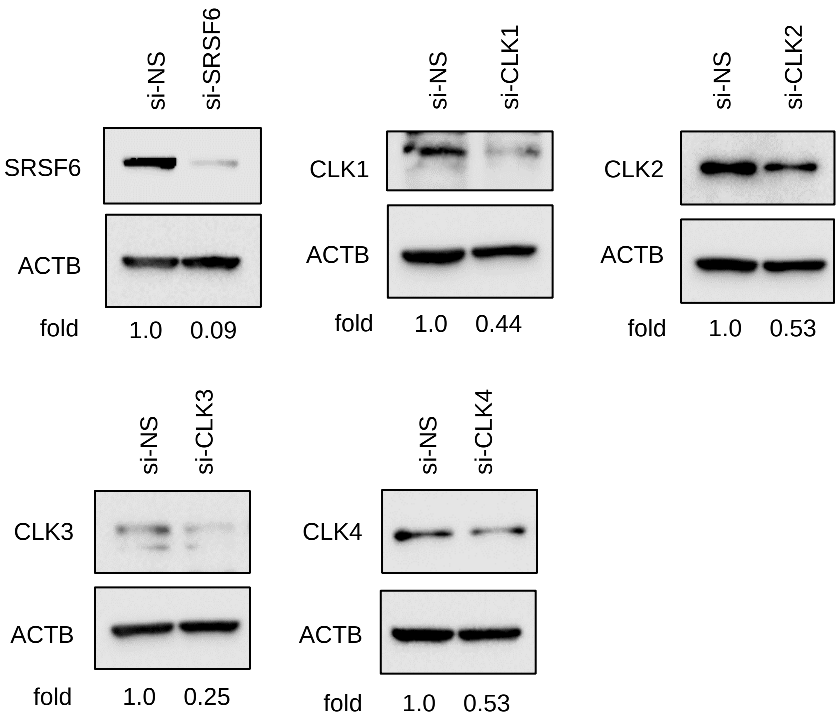

Supplementary Fig. 6. Western blot analysis for knockdown efficiency.

HeLa cells were transfected with non-targeting siRNA (si-NS) or siRNA targeting SRFS6, CLK1, CLK2, CLK3, and CLK4. Forty-eight hours after the transfection, each protein expression was analyzed by Western blot. Representative data from two experiments are shown. ACTB served as a loading control.

Supplementary Table 1. Summary of pre-clinical study of RECTAS.

Toxicity test		Species	Dose	Route[#1]	Result[#2]
General toxicity	Clinical sign or mortality<br>Body weight change<br>Necropsy check	Rat (n=5)	0, 100, 300, 1,000 mg/kg BW, single dose[#3]	p.o.	n/d
	Clinical sign or mortality<br>Body weight change<br>Necropsy check<br>Food consumption<br>Hematology test[#4]<br>(RBC, HGB, HCT, PLT, MCV, MCH, MCHC, WBC, RET, PT, APTT, Fbg, AST, ALT, ALP, T-Cho, TG, TP, IN, CRE, T-Bil, Glu, IP, Ca, Na, K, Cl)<br>Body weight, food consumption	Rat (n=3)	0, 40, 200, 1,000 mg/kg BW/day, 14 days	p.o.	n/d
Genotoxicity	Ames test			in vitro	negative
	Micronucleus test			in vitro	negative
Cell permiability	Cacl-2		10 µM	in vitro	95.5 ± 0.02%
Metabolic stability	Human liver microsome (0.5 mg/mg)		1 µM	in vitro	$T_{1/2}$ = 98.36 min

[#1]RECTAS was resuspended in 0.5% carboxymethyl cellulose for p.o.

[#2]n/d, no severe toxicity attributable to RECAS administration was detected.

[#3]observation was conducted for 14 days following the administration.

[#4]RBC, red blood cell count; HGB, hemoglobin; HCT, hematocrit; PLT, platelet; MCV, mean corpuscular volume; MCH, mean corpuscular hemoglobin; MCHC, mean corpuscular hemoglobin concentration; WBC, white blood cell count; RET, reticulocyte count; PT, prothrombin time; APTT, activated partial thromboplastin time; Fbg, fasting blood glucose; AST, aspartate aminotransferase; ALT, alanine aminotransferase; ALP, alkalne phosphatase; T-Cho, total cholesterol; TG, triglycerides; TP, total protein; UN, urea nitrogen; CRE, creatinine; T-Bil, total bilirubin; Glu, glucose; IP, inorganic phosphorus; Ca, calcium; Na, sodium; K, potassium; Cl, chloride.

Supplementary Table 2. List of primers used in this study.

Name	Target gene	Species	orientation	sequence
oAM13	*ACTB*	*H. Sapiens*	forward	5′-CCAACCGCGAGAAGATGACC-3′
oAM14	*ACTB*	*H. Sapiens*	reverse	5′-AGCTTCTCCTTAATGTCACG-3′
oAM138	*IKBKAP*	*H. Sapiens*	forward	5′-GGATTGTCACTGTTGTGC-3′
oAM139	*IKBKAP*	*H. Sapiens*	reverse	5′-CAAGTTAATATGATTCACAGAATCT-3′
HsIKAPRT18F	*IKBKAP*	*H. Sapiens*	forward	5’-TGTTTTTGCCTGAGGGATGC-3’
HsIKAPRT21R	*IKBKAP*	*H. Sapiens*	reverse	5’-AATGAAGGTTTCCACATTTCCA-3’
oAM669	*RBM24*	*H. Sapiens*	forward	5′-GTCTTCGGCGAGATCGAGGA-3′
oAM671	*RBM24*	*H. Sapiens*	reverse	5′-TGATCCTTGGTTTTGCTCCT-3′
oAM365	*Actb*	*H. Sapiens*	forward	5′-CCCAGAGCAAGAGAGGTATC-3′
oAM366	*Actb*	*H. Sapiens*	reverse	5′-GACGCAGGATGGCGTGAGG-3′
oAM666	*Ikbkap*	*M. Musculus*	forward	5′-TCACGGATCGTGACAGTTGT-3′
oAM667	*Ikbkap*	*M. Musculus*	reverse	5′-GGGTTATGGTCATGAATCAG-3′
Rbm24-F	*Rbm24*	*M. Musculus*	forward	5′-CTGCGCAAGTACTTTGAGGTCTTCG-3′
Rbm24-R	*Rbm24*	*M. Musculus*	reverse	5′-GTAGACGTAGTGGGCGGGG-3′
oAM124	GST of reporter vector	-	forward	5′-TCCAGCAAGTATATAGCAT-3′
oAM126	RFP of reporter vector	-	reverse	5′-CTCCTTGATGACGTCCTCG-3′
