# Supplementary material for: Therapeutic manipulation of IKBKAP mis-splicing with a small molecule to cure familial dysautonomia
Source: Nat Commun. 2021 Jul 23;12:4507. doi: 10.1038/s41467-021-24705-5 (PMC8302731; doi:10.1038/s41467-021-24705-5)
Supplement: Supplementary file 4 — Description of Additional Supplementary Files [file 41467_2021_24705_MOESM4_ESM.pdf]

Supplementary Data 1. Altered splice events following RECTAS treatment in FD patient fibroblasts.

Supplementary Data 2. Altered splice events in IKBKAP IVS20+6T>C transgenic mice DRG by RECTAS treatment (300 mg/kg BW).
