# Supplementary material for: Therapeutic manipulation of IKBKAP mis-splicing with a small molecule to cure familial dysautonomia
Source: Nat Commun. 2021 Jul 23;12:4507. doi: 10.1038/s41467-021-24705-5 (PMC8302731; doi:10.1038/s41467-021-24705-5)
Supplement: Supplementary file 5 — Reporting Summary [file 41467_2021_24705_MOESM5_ESM.pdf]

nature research

Corresponding author(s): Masatoshi Hagiwara

Last updated by author(s): Mar 12, 2021

Reporting Summary

Nature Research wishes to improve the reproducibility of the work that we publish. This form provides structure for consistency and transparency in reporting. For further information on Nature Research policies, see our Editorial Policies and the Editorial Policy Checklist.

Statistics

For all statistical analyses, confirm that the following items are present in the figure legend, table legend, main text, or Methods section.

n/a	Confirmed	
☐	☒	The exact sample size (*n*) for each experimental group/condition, given as a discrete number and unit of measurement
☐	☒	A statement on whether measurements were taken from distinct samples or whether the same sample was measured repeatedly
☐	☒	The statistical test(s) used AND whether they are one- or two-sided<br>*Only common tests should be described solely by name; describe more complex techniques in the Methods section.*
☒	☐	A description of all covariates tested
☒	☐	A description of any assumptions or corrections, such as tests of normality and adjustment for multiple comparisons
☐	☒	A full description of the statistical parameters including central tendency (e.g. means) or other basic estimates (e.g. regression coefficient) AND variation (e.g. standard deviation) or associated estimates of uncertainty (e.g. confidence intervals)
☐	☒	For null hypothesis testing, the test statistic (e.g. *F*, *t*, *r*) with confidence intervals, effect sizes, degrees of freedom and *P* value noted<br>*Give P values as exact values whenever suitable.*
☒	☐	For Bayesian analysis, information on the choice of priors and Markov chain Monte Carlo settings
☒	☐	For hierarchical and complex designs, identification of the appropriate level for tests and full reporting of outcomes
☒	☐	Estimates of effect sizes (e.g. Cohen's *d*, Pearson's *r*), indicating how they were calculated

Our web collection on statistics for biologists contains articles on many of the points above.

Software and code

Policy information about availability of computer code

Data collection	Detection of RT-PCR and Western blots was conducted with ChemiDoc MP Imaging System (Bio-Rad, Hercules, CA). Fluorescent signals from cells were collected with a BZ-X710 fluorescence microscope (Keyence, Osaka, Japan). Absorbance at 450 nm for cell proliferation was measured with ARVO X5 multimode plate reader (Perkin Elmer, Waltham, MA).
Data analysis	RT-PCR was analyzed with Image Lab software 6.0.1 (Bio-Rad). Western blot intensity was analyzed with Image J 1.52a (Schnerider CA et al. Nat. Methods, 2012). Fluorescent signals obtained through BZ-X710 fluorescence microscope were analyzed by hybrid cell count on BZ-X analyzer 1.4.0.1 (Keyence). Regression analysis for determination of EC50 and CT50 was conducted by GraphPad Prism 7.05. (GraphPad Software, San Diego, CA). Real Time Analysis (RTA) 1.9, STAR (2.4.1d), and Integrated Genomic Viewer (2.8.0) were used for RNA-Seq analysis. Chemical structure was drawn by ChemDraw 19.0 (Perkin Elmer).

For manuscripts utilizing custom algorithms or software that are central to the research but not yet described in published literature, software must be made available to editors and reviewers. We strongly encourage code deposition in a community repository (e.g. GitHub). See the Nature Research guidelines for submitting code & software for further information.

Data

Policy information about availability of data

All manuscripts must include a data availability statement. This statement should provide the following information, where applicable:

- Accession codes, unique identifiers, or web links for publicly available datasets
- A list of figures that have associated raw data
- A description of any restrictions on data availability

The original RNA-seq data were deposited at the National Bioscience Database Center database with the accession ID hum0180 for patient fibroblasts, and at the Gene Expression Omnibus of National Center for Biotechnology Information for transgenic mice with the accession ID GSE161109. Source data are provided with

this paper. Repbase database is available at https://www.girinst.org/repbase/. GRCh38 human reference genome and Ensemble annotation GRCh38.94 are available from Ensembl database (https://ensembl.org).

Field-specific reporting

Please select the one below that is the best fit for your research. If you are not sure, read the appropriate sections before making your selection.

- [x] Life sciences
- [ ] Behavioural & social sciences
- [ ] Ecological, evolutionary & environmental sciences

For a reference copy of the document with all sections, see nature.com/documents/nr-reporting-summary-flat.pdf

Life sciences study design

All studies must disclose on these points even when the disclosure is negative.

Sample size	Sample size for each experiment was indicated in legends. Sample size was chosen based on previous experimental experience with similar assays and/or sized generally employed in the field (references include Shibata S. et al. Cell Chem. Biol. (2020) 27(12):1472-1482; Ajiro M. et al. Nucleic Acids Res. (2016) 44(4): 1854-1870; Yoshida M. et al. Proc. Natl. Acad. Sci. USA (2015) 112(9):2764-2769). No statistical methods were employed to predetermine sample size.
Data exclusions	No data were excluded.
Replication	Replication study was conducted with repeat number indicated in each figure legends, and all attempts were successful.
Randomization	All samples used in this study, including transgenic mice and cultured cells, were allocated randomly to each condition.
Blinding	Evaluation for fluorescence intensities from microscopic analysis of splicing reporter assay was conducted in a blind manner. For RT-PCR, Western blot, and biochemical assay, cell or compound types were known when prepare the samples or set up the assay.

Reporting for specific materials, systems and methods

We require information from authors about some types of materials, experimental systems and methods used in many studies. Here, indicate whether each material, system or method listed is relevant to your study. If you are not sure if a list item applies to your research, read the appropriate section before selecting a response.

Materials & experimental systems

n/a	Involved in the study	
[ ]	[x]	Antibodies
[ ]	[x]	Eukaryotic cell lines
[x]	[ ]	Palaeontology and archaeology
[ ]	[x]	Animals and other organisms
[x]	[ ]	Human research participants
[x]	[ ]	Clinical data
[x]	[ ]	Dual use research of concern

Methods

n/a	Involved in the study	
[x]	[ ]	ChIP-seq
[x]	[ ]	Flow cytometry
[x]	[ ]	MRI-based neuroimaging

Antibodies

Antibodies used

anti-U1-70k mouse monoclonal antibody (9C4.1) (#05-1588, Merk Millipore, Burlington, MA) 1:500
anti-SmB/B′ mouse monoclonal antibody (Y12) (#MA5-13449, Thermo Fisher Scientific, Waltham, MA) 1:500
anti-CLK1 rabbit polyclonal antibody (#ARP52021_P050, Aviva systems biology, San Diego, CA) 1:500
anti-CLK2 rabbit polyclonal antibody (#ab65082, abcam, Cambridge, UK) 1:500
anti-CLK3 rabbit polyclonal antibody (#3256, Cell Signaling Technology, Danvers, MA) 1:500
anti-CLK4 rabbit polyclonal antibody (#ab104321, abcam, Cambridge, UK) 1:500
anti-β-actin mouse monoclonal antibody (Ac-15) (#sc-69879, Santa Cruz Biotechnology, Dallas, TX) 1:4,000
anti-SR protein (1H4G7) mouse monoclonal antibody (#33-9400, Thermo Fisher Scientific, Waltham, MA) 1:200
anti-Lamin B1 (EPR8985) rabbit monoclonal antibody (#ab133741, abcam, Cambridge, UK) 1:500
anti-OCT4 rabbit monoclonal antibody (T.631.9) (#MA5-14845, Thermo Fisher Scientific, Waltham, MA) 1:400
anti-SSEA4 mouse monoclonal antibody (MC-813-70) (#MA1-021, Thermo Fisher Scientific, Waltham, MA) 1:500
anti-SOX10 rabbit monoclonal antibody (EPR4007) (#ab155279, abcam, Cambridge, UK) 1:250
anti-BRN3A mouse monoclonal antibody (5A3.2) (#MAB1585, Merck Millipore, Burlington, MA) 1:200
anti-beta III tubulin (TUBB3) rabbit monoclonal antibody (EP1569Y) (#ab52623, abcam, Cambridge, UK) 1:500

Validation

anti-U1-70k mouse monoclonal antibody (9C4.1) (#05-1588, Merk Millipore, Burlington, MA)
(https://www.merckmillipore.com/JP/ja/product/Anti-U1-70K-Antibody-clone-9C4.1,MM_NF-05-1588)

anti-SmB/B' mouse monoclonal antibody (Y12) (#MA5-13449, Thermo Fisher Scientific, Waltham, MA)
'(https://www.thermofisher.com/antibody/product/SNRPB-Antibody-clone-Y12-Monoclonal/MA5-13449)

anti-CLK1 rabbit polyclonal antibody (#ARP52021_P050, Aviva systems biology, San Diego, CA)
(https://www.avivasysbio.com/clk1-antibody-n-terminal-region-arp52021-p050.html)

anti-CLK2 rabbit polyclonal antibody (#ab65082, abcam, Cambridge, UK)
(https://www.abcam.co.jp/clk2-antibody-ab65082.html)

anti-CLK3 rabbit polyclonal antibody (#3256, Cell Signaling Technology, Danvers, MA)
(https://www.cellsignal.com/products/primary-antibodies/clk3-antibody/3256)

anti-CLK4 rabbit polyclonal antibody (#ab104321, abcam, Cambridge, UK)
(https://www.abcam.co.jp/clk4-antibody-ab104321.html)

anti-β-actin mouse monoclonal antibody (Ac-15) (#sc-69879, Santa Cruz Biotechnology, Dallas, TX)
(https://www.scbt.com/ja/p/beta-actin-antibody-ac-15)

anti-SR protein (1H4G7) mouse monoclonal antibody (#33-9400, Thermo Fisher Scientific, Waltham, MA)
(Takeshi Fukuhara, et al. PNAS (2006) doi/10.1073/pnas.0604616103)

anti-Lamin B1 (EPR8985) rabbit monoclonal antibody (#ab133741, abcam, Cambridge, UK)
(https://www.abcam.co.jp/lamin-b1-antibody-epr8985b-ab133741.html)

anti-OCT4 rabbit monoclonal antibody (T.631.9) (#MA5-14845, Thermo Fisher Scientific, Waltham, MA)
(https://www.thermofisher.com/antibody/product/OCT4-Antibody-clone-T-631-9-Monoclonal/MA5-14845)

anti-SSEA4 mouse monoclonal antibody (MC-813-70) (#MA1-021, Thermo Fisher Scientific, Waltham, MA)
(https://www.thermofisher.com/antibody/product/SSEA4-Antibody-clone-MC-813-70-Monoclonal/MA1-021)

anti-SOX10 rabbit monoclonal antibody (EPR4007) (#ab155279, abcam, Cambridge, UK)
(https://www.abcam.co.jp/sox10-antibody-epr4007-ab155279.html)

anti-BRN3A mouse monoclonal antibody (5A3.2) (#MAB1585, Merck Millipore, Burlington, MA)
(https://www.merckmillipore.com/JP/ja/product/Anti-Brn-3a-Antibody-POU-domain-protein-clone-5A3.2,MM_NF-MAB1585)

anti-beta III tubulin (TUBB3) rabbit monoclonal antibody (EP1569Y) (#ab52623, abcam, Cambridge, UK)
(https://www.abcam.co.jp/beta-iii-tubulin-antibody-ep1569y-ab52623.html)

Eukaryotic cell lines

Policy information about cell lines

Cell line source(s)

HeLa cells: Japanese Collection of Research Bioresources Cell Bank of the National Institutes of Biomedical Innovation, Health and Nutrition (Osaka, Japan)
Neuro 2A: Japanese Collection of Research Bioresources Cell Bank of the National Institutes of Biomedical Innovation, Health and Nutrition (Osaka, Japan)
Familial dysautonomia primary patient fibroblasts, homozygous for IKBKAP IVS20+6T>C GM02342 (P1): Coriell Institute (Camden, NJ, USA)
Familial dysautonomia primary patient fibroblasts, homozygous for IKBKAP IVS20+6T>C GM00850 (P2): Coriell Institute (Camden, NJ, USA)
Primary fibroblasts from healthy donor (TIG-114, C1): Japanese Collection of Research Bioresources Cell Bank of the National Institutes of Biomedical Innovation, Health and Nutrition (Osaka, Japan)
Primary fibroblasts from healthy donor (TIG-108, C2): Japanese Collection of Research Bioresources Cell Bank of the National Institutes of Biomedical Innovation, Health and Nutrition (Osaka, Japan)
Healthy donor control of induced-pluripotent stem cells (C1): established in Nadia Zeltner et al. Nat. Med. (2016) 22(12): 1421-1427
Induced-pluripotent stem cells from familial dysautonomia patient (M1): established in Nadia Zeltner et al. Nat. Med. (2016) 22(12): 1421-1427

Authentication

HeLa cells: cell identity was confirmed by STR profiling analysis.
Neuro 2A: Cells obtained from the provider was directly used in experiments. No further authentification was performed.
Familial dysautonomia primary patient fibroblasts, homozygous for IKBKAP IVS20+6T>C GM02342 (P1): Cells obtained from the provider was directly used in experiments. No further authentification was performed.
Familial dysautonomia primary patient fibroblasts, homozygous for IKBKAP IVS20+6T>C GM00850 (P2): Cells obtained from the provider was directly used in experiments. No further authentification was performed.
Primary fibroblasts from healthy donor (TIG-114, C1): Cells obtained from the provider was directly used in experiments. No further authentification was performed.
Primary fibroblasts from healthy donor (TIG-108, C2): Cells obtained from the provider was directly used in experiments. No

	further authentification was performed.<br>Healthy donor control of induced-pluripotent stem cells (C1): Cells established in Nadia Zeltner et al. Nat. Med. (2016) 22(12): 1421-1427 were directly applied to this study.<br>Induced-pluripotent stem cells from familial dysautonomia patient (M1): Cells established in Nadia Zeltner et al. Nat. Med. (2016) 22(12): 1421-1427 were directly applied to this study.
Mycoplasma contamination	Micoplasma tests by qPCR are performed on a regular basis and all cells were confirmed negative.
Commonly misidentified lines<br>(See ICLAC register)	No commonly misidentified cell lines were used in the study.

Animals and other organisms

Policy information about studies involving animals; ARRIVE guidelines recommended for reporting animal research

Laboratory animals	Transgenic mouse strain, introduced with human IKBKAP (IVS20+6T>C mutant) genomic sequence from mutated BAC clone, was previously established (Hims M.M. et al. Genomics (2007) 90:389-396), and obtained from Drs. Pickel and Slaugenhaupt. Mice hemizygously harbor human IKBKAP (IVS20+6T>C), but are asymptomatic due to presence of endogenous Ikbkap homologue. Mice were maintained in animal facility of Cold Spring Harbor Laboratories, and those more than eight-week old were used in this study. Sex of each group of mice are as following; male (n=2) for no treatment, male (n=3) and female (n=3) for CMC administration, male (n=3) and female (n=2) for 300 mg/kg BW RECTAS administration, and male (n=2) and female (n=1) for 400 mg/kg BW RECTAS administration.
Wild animals	No wild animals were used in the study.
Field-collected samples	No field collected samples were used in the study.
Ethics oversight	All mouse experiments were approved by Cold Spring Harbor Laboratories.

Note that full information on the approval of the study protocol must also be provided in the manuscript.
